# Synesthesia does not help to recover perceptual dominance following flash suppression

**DOI:** 10.1038/s41598-021-87223-w

**Published:** 2021-04-07

**Authors:** Diana Jimena Arias, Dave Saint-Amour

**Affiliations:** 1 Department of Psychology, Université du Québec à Montréal, Montreal, H2X 3P2 Canada; 2 Cognitive Neurosciences Research Center, Université du Québec à Montréal, Montreal, H2X 3P2 Canada; 3 Research Center of the Sainte-Justine University Hospital, Montreal, H3T 1C5 Canada

**Keywords:** Consciousness, Perception, Sensory processing

## Abstract

Grapheme-colour synesthesia occurs when letters or numbers elicit an abnormal colour sensation (e.g., printed black letters are perceived as coloured). This phenomenon is typically reported following explicit presentation of graphemes. Very few studies have investigated colour sensations in synesthesia in the absence of visual awareness. We took advantage of the dichoptic flash suppression paradigm to temporarily render a stimulus presented to one eye invisible. Synesthetic alphanumeric and non-synesthetic stimuli were presented to 21 participants (11 synesthetes) in achromatic and chromatic experimental conditions. The test stimulus was first displayed to one eye and then masked by a sudden presentation of visual noise in the other eye (flash suppression). The time for an image to be re-perceived following the onset of the suppressive noise was calculated. Trials where there was no flash suppression performed but instead mimicked the perceptual suppression of the flash were also tested. Results showed that target detection by synesthetes was significantly better than by controls in the absence of flash suppression. No difference was found between the groups in the flash suppression condition. Our findings suggest that synesthesia is associated with enhanced perception for overt recognition, but does not provide an advantage in recovering from a perceptual suppression. Further studies are needed to investigate synesthesia in relation to visual awareness.

## Introduction

Synesthesia is a perceptual phenomenon in which a stimulus elicits an abnormal and concurrent sensation in the same or a different sensory modality. Among the different types of synesthesia, grapheme-colour synesthesia is relatively common and it consists of colour perceptions evoked by grey scale alphanumeric images (letters and/or digits)^1^. Synesthetic associations are consistent over time^2–4^ and are automatic or difficult to discard when elicited^3,5–9^.

Grapheme-colour synesthesia has been most frequently investigated through the explicit presentation of synesthetic graphemes or digits, as shown with stroop-like tasks and visual search paradigms^5–10^. However, synesthesia remains much less studied when observers are unaware of a stimulus. For a review see^11^. Mattingley et al.^9^ adapted a masking paradigm in order to test synesthetic stimuli under conditions preventing their visibility. They measured how long it takes for participants to name the colour of a target mask that precedes the presentation of a letter prime, which evokes a synesthetic colour, either congruent or incongruent with the colour of the target mask. Congruent and incongruent trials were used to assess the synesthetic interference effect on naming the target’s colour. The letter prime was visible when presented for 500 ms and invisible when presented for 56 ms or less. The synesthetic interference effect was observed only when the prime was visible to participants, i.e., this effect disappeared when the letter did not access visual awareness. Furthermore, synesthete participants showed implicit priming effects similar to controls for non-synesthetic inducing stimuli; that is, the brief presentation of a semantic prime (e.g., an upper-case letter “A”) improved the subsequent recognition of a congruent target stimulus (e.g., lower-case letters “a”).

Synesthesia was also tested using the attentional blink paradigm in which two successive targets, T1 and T2, are presented in a sequence and separated by distractors (masks)^12^. In this paradigm, the second target T2 is rendered invisible when the presentation time between both targets does not exceed the attentional window, which is between 300 and 500 ms. Rich et al.^13^ presented a synesthetic prime (T2) within and outside of the attentional window. A colour probe was presented at the end of the attentional blink sequence. The prime elicited a synesthetic colour that was either congruent or incongruent with the colour probe, producing an interference effect in the colour naming of the probe. Albeit modest, a reliable interference effect was found when the prime was visible. No interference effect was obtained when the prime was presented within the temporal window of the attentive blink. In another study using the same attentional blink design, however, some synesthetic participants (5 out of 10) were able to perceive synesthetic colours, even when the synesthetic primes fell within the attentional blink temporal window^14^. As such, an interference effect in a colour naming task was still noticed when participants were unable to overtly identify the synesthetic prime in the visual sequence. In line with this finding, it has been reported in four grapheme-colour synesthetes that conscious letter recognition is not required to perceive the colour of hidden letters^15^.

The aforementioned studies are not conclusive with regard to whether synesthesia can occur without visual awareness^9,13–15^. To further investigate this question, we looked at the flash suppression paradigm, which has been used extensively to study visual processing in the absence of awareness^16,17^. The flash suppression phenomenon is typically induced by two different monocular images presented asynchronously; an image is first presented to one eye for a while (while a blank field is presented to the other eye), after which the second image is abruptly shown, i.e., flashed, to the other eye at the corresponding retinal points. Unlike the masking and attentional blink paradigms, flash suppression allows a stimulus to be rendered invisible temporarily, even though it remains physically present for the observer. Thus, it allows manipulation of the onset of interocular suppression, i.e., the awareness of the suppressed stimulus, before binocular rivalry between the competing images occurs^16^. Unlike binocular rivalry, which involves alternation of perceptual dominance and complex neural dynamics at several brain levels^18^, flash suppression offers better control of the monocular suppression^19^. Previous studies using flash suppression have shown that even if subjects are not aware of the presence of a stimulus in one eye, visual processing of that stimulus may still occur^16,20–22^.

It was previously reported in normal observers that the flash suppression of a coloured Gabor grating is shorter than that of an achromatic Gabor grating^17^, and colour can break suppression more efficiently than other visual features^23^. Thus the present study tested the hypothesis that the effect of flash suppression on the synesthetic (subjectively colored) stimulus is weaker than that of the non-synesthetic stimulus. By measuring the time the hidden stimulus takes to break through to awareness, we aimed to determine the effect of synaesthesia on perceptual suppression. If synesthesia occurs when the participants are not aware of the synesthetic achromatic grapheme, the prediction is that they will exhibit a shorter duration of suppression in comparison to non-synesthetic conditions.

## Results

One synesthete (participant 6) consistently reported longer reaction times (RTs) for the non-flash-suppression trials, significantly longer than those obtained from the rest of the participants (Z-scores ≤ − 3.5). This participant was thus excluded from the data analysis.

**Figure 1:**
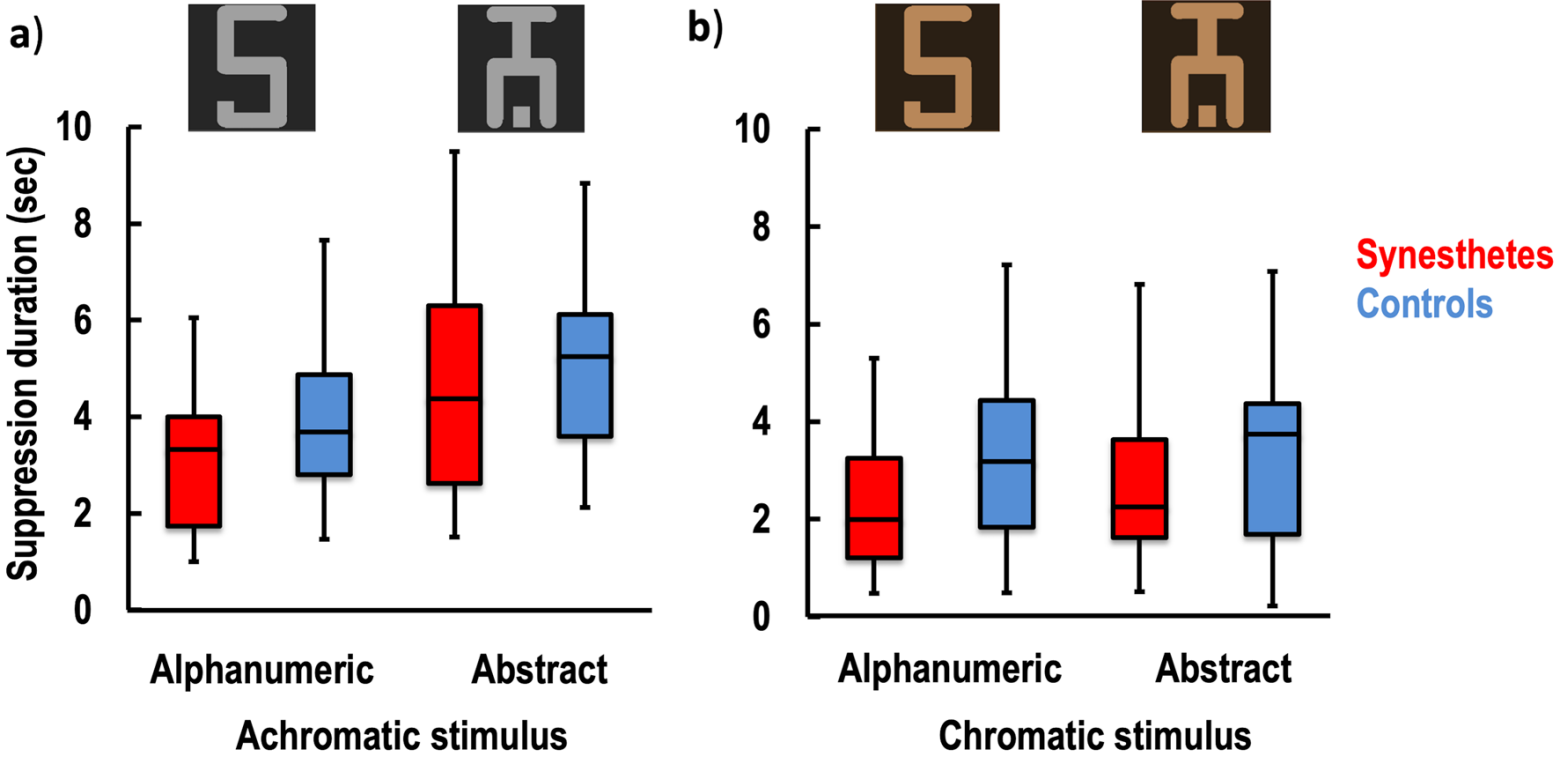
Perceptual stimulus suppression. The flash suppression duration is shown for the achromatic (**a**) and chromatic (**b**) stimulus conditions in the synesthetic participants (red) and the control participants (blue). Considering that the physical achromatic grapheme stimulus is associated with a colored percept in the synesthetes, a significant difference between the alphanumeric and abstract stimuli in the achromatic condition was expected in the synesthetes, but not in the controls. The chromatic condition is illustrated by the orange colour as an example. The whiskers indicate the minimum and maximum range of the distributions; the top and bottom of the boxes show the first and third quartiles (the 25th and 75th percentiles), and the horizontal bars inside the boxes represent the medians.

The analysis of variance (ANOVA) conducted on the perceptual flash suppression trials showed no main group effect [*F*_(1,19)_ = 0.523, *p* = 0.478, η^2^_p_ 0.027] or interaction of the group with the other factors: condition*group [*F*_(1,19)_ = 0.280, *p* = 0.603, η^2^_p_ 0.015], stimulus*group [*F*_(1,19)_ = 0.716, *p* = 0.408, η^2^_p_ 0.036] and the condition*stimulus*group [*F*_(1,19)_ = 0.001, *p* = 0.975, η^2^_p_ 0.00005]. However, a main effect of the condition [*F*_(1,19)_ = 63.833, *p* < 0.001, η^2^_p_ = 0.771], the stimulus [*F*_(1,19)_ = 21.591, *p* < 0.001, η^2^_p_ = 0.532] as well as the interaction of condition*stimulus [*F*_(1,19)_ = 36.989, *p* < 0.001, η^2^_p_ = 0.661] was found to be statistically significant. As is depicted in Fig. 1, all participants exhibited a significantly shorter duration of suppression in the chromatic condition than in the achromatic condition, and this effect was stronger for alphanumeric stimuli.

Two sensitivity analyses were also performed. First, an additional ANOVA was conducted on the perceptual suppression duration while excluding the participant reporting a “d” synesthetic stimulus, instead of the "5" observed by the other synesthetes. Second, a similar approach was conducted by excluding the synesthete participant who showed a projector synesthesia profile, as revealed by the online synesthesia battery test. Results from these two ANOVA remained the same (data not shown). It should be noted that *Z*-score tests revealed that the performance of the projector synesthete participant was not significantly different from the other synesthete participants, or from the control participants.

**Figure 2:**
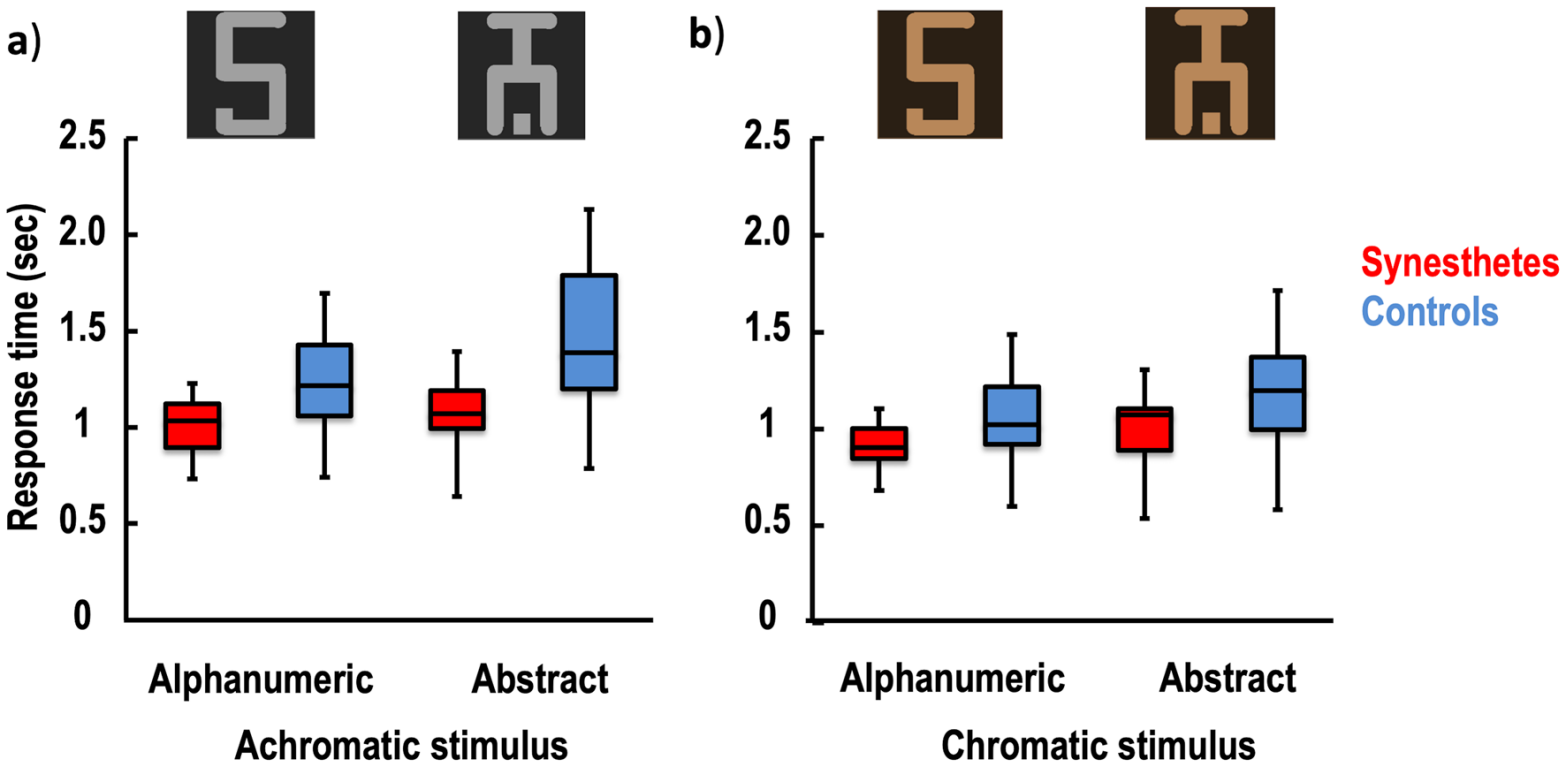
Stimulus detection in the absence of flash suppression. Response time is shown for the achromatic (**a**) and chromatic (**b**) stimulus conditions in the synesthetic participants (red) and the control participants (blue). Considering that the physical achromatic grapheme stimulus is associated with a colored percept in the synesthetes, a significant difference between the alphanumeric and abstract stimuli in the achromatic condition was expected in the synesthetes, but not in the controls. The chromatic condition is illustrated by the orange colour as an example. The whiskers indicate the minimum and maximum range of the distributions, the top and bottom of the boxes show the first and third quartiles (the 25th and 75th percentiles), and the horizontal bars inside the boxes represent the medians.

For non-flash-suppression trials, the ANOVA revealed robust significant effects of the condition [*F*_(1,19)_ = 21.111, *p* < 0.001, η^2^_p_ = 0.526], the stimulus [*F*_(1,19)_ = 23.970, *p* < 0.001, η^2^_p_ = 0.558], and the group [*F*_(1,19)_ = 5.651, *p* = 0.028, η^2^_p_ = 0.229]. No interaction effects between factors were found to be statistically significant: condition*stimulus*group [*F*_(1,19)_ = 2.268, *p* = 0.146, η^2^_p_ = 0.107], condition*stimulus [*F*_(1,19)_ = 0.586, *p* = 0.453, η^2^_p_ = 0.030], condition*group [*F*_(1,19)_ = 2.644, *p* = 0.120, η^2^_p_ = 0.122], stimulus*group [*F*_(1,19)_ = 2.877, *p* = 0.106, η^2^_p_ = 0.132]. Thus, participants perceived chromatic stimuli faster than achromatic stimuli (Fig. 2). In addition, alphanumeric stimuli were more rapidly reported than abstract stimuli. In comparison to controls, target detection in all conditions was in general faster in synesthete participants. Of note, the performance of the synesthetes in the achromatic condition was not statistically different (*t*_(9)_ = 1.231, *p* = 0.249) between the alphanumeric (M = 1.001, SD = 0.164) and abstract stimuli (M = 1.077 SD = 0.216).

To confirm these findings with a maximum of statistical power, data were also analyzed using mixed effects modeling to treat subjects as a random factor, and the condition, stimulus and group as fixed factors. This approach was applied separately for flash suppression trials and non-flash-suppression trials. Results (see Supplementary Tables S1 and S2) were in line with those obtained with the aforementioned ANOVAs. However, the mixed model in the non-flash suppression condition (Table S2) revealed a trend toward significance for the interaction between group and condition (*p* = 0.059). Exploratory follow-up analysis indicated that synesthetes were actually faster than the controls in the achromatic condition (M = 1.039, SD = 0.080 vs. M = 1.347, SD = 0.077, *p* = 0.011).

Bayesian repeated measures ANOVAs were also conducted to quantify the plausibility of both the null and alternative hypotheses, permitting interpretation of null findings. In Bayesian inference, the likelihood of the data is considered under both hypotheses, and these probabilities are compared via the Bayes factor (BF). According to Lee and Wagenmakers’ classification^24^, the level of evidence was deemed inconclusive/anecdotal for BF between 0.33 and 3, moderate for BF < 0.33 or > 3, and strong for BF < 0.1 or > 10. Following the JASP guidelines^25^, BF comparing the null model against all other models were computed and each experimental effects was obtained by calculating the inclusion BF across matched models. Results revealed overwhelming evidence in favor of a main effect of condition (BF of 1.887e+8 and 8.465e+3 for flash and non-flash trials, respectively), a main effect of stimulus (BF of 8892.142 and 3226.375 for flash and non-flash trials, respectively) as well as their interaction (BF = 17.523, for flash trials). However, inconclusive evidence was found for or against of a main or interaction effect of group (all BF values ranged between 0.33 and 3). Details are presented in Supplementary Tables S3 and S4.

## Discussion

The present study investigated whether synesthesia shortens the duration of interocular suppression. Results from the flash suppression trials revealed no evidence of suppression modulation from synesthetic stimuli, and no significant differences between the synesthete and non-synesthete groups. However, chromatic stimuli exhibited shorter suppression latencies, as they emerge more quickly, than achromatic stimuli. We also found in both synesthetes and controls that, in comparison to the abstract stimuli, the alphanumeric stimuli reduced suppression durations. In the non-flash-suppression trials, all participants detected coloured stimuli faster than achromatic stimuli. They also showed shorter reaction times for the detection of alphanumeric stimuli than for abstract stimuli. In the non-flash-suppression trials, we found that the synesthete participants exhibited a better performance than the controls, regardless of the type (synesthetic or not) and the colour (chromatic or achromatic) of the stimuli used. The small sample size is a major limitation of the study, which might be particularly challenging with between-subject designs and non-significant findings. We statistically addressed this issue by re-analyzing the data using mixed effects models, with the subject level variability as a random effect. The results remained the same. Bayesian analyses support the presence of strong effects of stimulus chromaticity and stimulus type on performance, but did not provide sufficient evidence for or against an effect of synesthesia, i.e., BF > 0.33 and < 3. Thus, given the modest magnitude of the Bayes factors, it appears that the observed data were insensitive to detect an effect of synesthesia, suggesting that our study was underpowered (i.e., more participants might be required).

The stimulus features, i.e., the colour and familiarity, was found to influence the performance in all participants, whether under interocular suppression viewing condition or not. Some data from normal observers in the seminal study of Tsuchiya and Koch^17^ on the continuous flash suppression (CFS) suggest a break through effect of chromaticity that favor stimulus predominance. This observation is in agreement with the notion that the interocular suppression of colour is weaker than on form^23^, and with the general consensus that colour improves signal detection by enhancing the saliency of the stimulus^26,27^. Regarding the effect of the alphanumeric stimuli, it is possible that response time in detection (with or without interocular suppression) was shorter because of higher familiarity and/or meaning of those stimuli, in comparison to the abstract and unusual symbols. Although it is debated whether semantic processing under continuous flash suppression occurs at all (see^28,29^), Gobbini and coworkers^30^ reported that processing of familiar stimuli (faces) is more likely to resist flash suppression than non-familiar ones. This result is in line with the fact that, under explicit viewing conditions, familiar stimuli are more efficiently processed and detected than meaningless or unfamiliar targets^31^. Interestingly, our results also showed that the effect of stimulus colour and familiarity on reducing interocular suppression are not independent, but interact. Thus, coloured stimuli overcame suppression faster when they were also familiar.

The current study failed to demonstrate that synesthetic stimuli biased the performance of the synesthete, suggesting that synesthesia does not bias interocular suppression or modulate stimuli that are rendered temporarily invisible by flash suppression. This interpretation is in agreement with the notion that conscious recognition is required in order to elicit synesthetic percepts^9^, including the study of Rich and Mattingley^13^ using attentional blink, in which no synesthetic effect (i.e., interference stroop-like effect of T2 on subsequent colour naming) was observed when the synesthetic prime (T2) was not consciously perceived. However, some methodological aspects of our study may have prevented any breakthrough effects of synesthesia. Indeed, small target size (1° × 0.6°) was used to minimize piecemeal percepts (and thus perceptual ambiguity^32^). Based on our pilot experiments that confirmed that targets break through into awareness as an exclusive percept most of time, we asked the participants to respond when the stimulus re-appeared in its entirety. However, we cannot exclude the possibility that some piecemeal perception may have occurred. Thus, a more liberal instruction (e.g., respond as soon as you feel you can identify the stimulus) could have yielded different results. In line with Hong and Blake^23^, one can speculate that some synesthetic percepts would have been reported in a piecemeal and unbound fashion before explicit detection of the stimulus.

The absence of bias to synesthetic stimuli was also observed for the viewing condition exempted from interocular suppression. Indeed, response time for synesthetes was not statistically faster for synesthetic stimuli than non-synesthetic stimuli in the achromatic condition (although the difference was trending). Based on the facilitator influence of colour on target detection^27^, we expected the achromatic synesthetic stimuli to evoke colour sensation, as opposed to non-synesthetic stimuli, to improve explicit visual detection. In line with our finding, some studies, under the explicit viewing condition, have reported that achromatic synesthetic stimuli do not always show a significant advantage in reaction time over achromatic non-synesthetic symbols^33,34^. Other studies have failed to find perceptual differences between synesthetes and non-synesthetes. For example, no advantage for synesthetes has been found in some visual search tasks^35,36^, or in the identification of embedded figures^37^. Furthermore, the putative brain atypicalities in synesthetes have been recently challenged^38–40^, and many brain mechanisms observed in synesthetes are likely to follow the same rules as those found in non-synesthete individuals^41,42^.

In the explicit detection task (Fig. 5), synesthetes were faster than controls, regardless of whether the stimuli were coloured and/or synesthetic. One parsimonious explanation is that synesthetes are better at detecting the types of visual stimulus. There is indeed some experimental evidence suggesting that individuals experiencing coloured synesthesia show atypical visual processing for non-synesthetic stimuli. For instance, synesthetes show superior colour perception compared to controls, not only for hue discrimination^43^ but also for luminance and chroma^44^. Moreover, lower contrast discrimination thresholds and enhanced performance in colour and shape/curvature discrimination tasks have been reported^45,46^. Other factors may also explain the performance of synesthetes. It has been suggested that synesthetes might exhibit some specific personality traits such as openness or disposition to get involved in new experiences, and that they are even more sensitive to mental imagery^47,48^. One can reasonably speculate that such subjective particularities might influence perception. Thus, synesthetes might differ in how willing they are to affirm the existence of a stimulus, which corresponds with the decision criterion in the signal detection theory, and impacts the observers’ discrimination responses. The faster target detection may therefore be the result of a criterion shift (response bias) rather than a true effect of discriminability. While our study was not designed to verify this possibility, it appears unlikely because the performance of the synesthetes was not different from the controls in the flash suppression condition. In addition, many studies using a signal detection theory design failed to reveal a significant difference in response bias between synesthete and non-synesthete participants^49–51^.

Further individual differences in experiencing synesthesia may have influenced the findings of the present study. It is well known that synesthetic experiences differ qualitatively between individuals. For instance, some synesthetes perceive colours as being “outside” of their body, while others perceive them internally, i.e., in the “mind’s eye”. These phenomenological distinctions in synesthetic percepts are known as the projector type and the associator type, respectively^10^, although this classification is still under debate^35,52^. The enhanced performance in synesthesia reported by most studies is best shown for projector synesthetes^10,53,54^, including for brain activity^55,56^. For example, the study conducted by Ramachandran et al.^15^ suggested that conscious letter recognition is not required for synesthetic perception, but only projector synesthetes were examined. By contrast, all synesthetes in the present study were classified as associator types, based on the self-report in the Synesthesia Battery, with the exception of one participant (ID2 in Table 1) with a score of 0.09, i.e., theoretically within the projector zone. Considering that this value was very close to zero (i.e., the associator/projector cutoff) and that her performance in all tasks was not significantly different from the other synesthetes, it is impossible to ascertain whether or not this participant was a true projector. For now, the role of such individual differences in the synesthetic experience remain poorly understood, as there is currently no reliable tool to distinguish projector from associator. In fact, most studies have failed to systematically evaluate and compare participants’ performance with regard to their synesthetic profiles^9,13–15^.

**Table 1:** Demographic and synesthesia characteristics of the synesthetic participants.

Demographic characteristics			Synesthesia characteristics			
Synesthetes	Age (year: month)	Sex	Consistency score	Projector/associator score		Alphanumeric stimulus
1	32:11	F	0.91	− 0.60	Associator	5 (red)
2	32:06	F	0.73	0.09	Projector	5 (blue)
3	23:11	F	0.43	− 0.17	Associator	5 (red)
4	24:10	F	0.65	− 1.75	Associator	5 (red)
5	22:03	M	0.49	− 1.92	Associator	5 (fuchsia)
6	25:00	F	0.53	− 1.83	Associator	5 (orange)
7	27:00	M	0.85	− 2.33	Associator	5 (red)
8	21:04	F	0.80	− 1.42	Associator	5 (green)
9	28:10	F	0.51	− 0.83	Associator	5 (green)
10	31:07	M	0.73	− 2.75	Associator	d (blue)
11	23:07	M	0.42	− 0.57	Associator	5 (orange)

Consistency and projector/associator scores were obtained from the Online Synesthesia Battery.

In summary, our results showed that synesthesia does not bias perceptual flash suppression, indicating that synesthesia is less likely to manifest under implicit conditions; however, a more comprehensive assessment of synesthesia in relation to visual awareness is necessary to support this interpretation. Further studies using different designs such as the continuous flash suppression paradigm, which does not require pre-adaptation to achieve reliable disappearance, are needed to directly test whether synesthesic processing can occur without conscious awareness of the stimulus. Some key individual difference variables also needed to be considered, such as atypical visual functioning or discrimination thresholds, synesthesia type (associator vs. projector), and subject's criterion.

## Methods

### Participants

Eleven grapheme-colour type synesthetes (Table 1) and 11 control participants were recruited to take part in this experiment. Synesthetes were matched with controls based on age (21–32 years old) and sex (4 men, 7 women). Grapheme-colour associations in the synesthetes were assessed qualitatively during a semi-structured interview and quantitatively using the grapheme-colour consistency test from the online Synesthesia Battery, developed by Eagleman^53^. The synesthete participants were assessed twice using this battery, with a minimum lapse of 2 months between each testing session. Consistency test scores (see Table 1) below 1.0 are indicators of synesthetic associations. Scores between 1.0 and 2.0 are not sufficiently conclusive to consider the presence of synesthetic associations, while scores higher than 2.0 rule out the possibility altogether. In our study, the average consistency scores for synesthetic participants between the initial testing and the re-testing were within the range of synesthetic associations. More precisely, the minimal average score for consistency was 0.43, while the maximum average score for consistency was 0.91. These values confirmed that the synesthetic associations reported by the participants were highly consistent over time. A projector-associator score to characterize the type of synesthetic association was also obtained from the Synesthesia Battery (Table 1). Negative scores indicate that the synesthesia percept is in the "mind’s eye" while positive scores indicate an “out of mind” synesthetic perception.

None of the participants had a history of neurological or psychiatric disorders, and reported normal or corrected-to-normal vision. Visual acuity was measured using the Snellen acuity chart and the contrast sensitivity FACT test (Stereo Optical Company Inc., Chicago, IL, United States). Stereoscopic vision was assessed using the Randot Test (Stereo Optical Co., Inc., Chicago, IL). All participants gave written informed consent before participating in this study. They also received a financial compensation ($20 CAN). The experimental procedure conformed to the World Medical Association’s Declaration of Helsinki and was approved by the Research Ethics Committee of the Université du Québec à Montréal (FSH-2013-92).

### Stimuli and design

**Figure 3:**
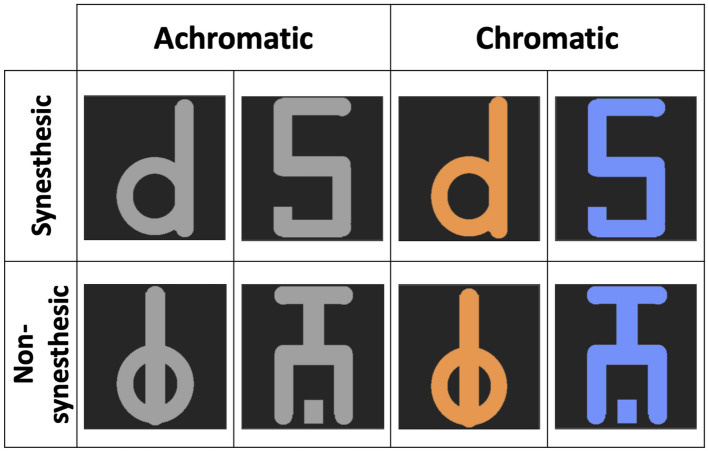
Stimuli. The synesthetic alphanumeric (first row) and the non-synesthetic abstract stimuli (second row) were randomly presented. The achromatic alphanumeric stimuli were experienced as colored for the synesthetes.

**Figure 4:**
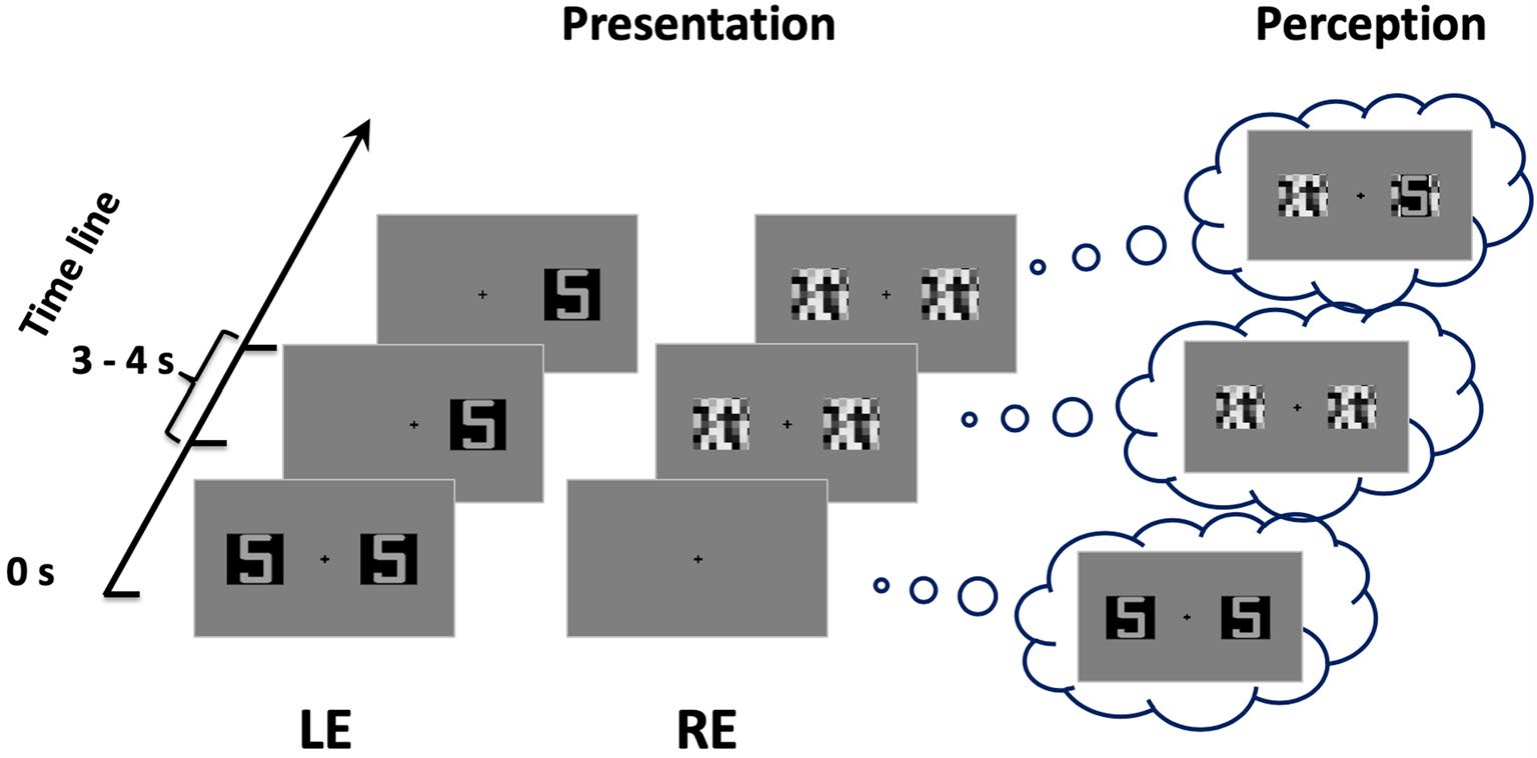
An example of a flash suppression trial. Following the noise patch stimulation in one eye, the initial pair of images in the opposite eye disappeared and one of the two images (either on the right or left side) reappeared after some time. LE: left eye; RE: right eye.

Three types of stimulus were used: a synesthetic stimulus, a non-synesthetic stimulus, and a suppressor stimulus. The synesthetic stimulus was a number “5” for all participants except one (see Table 1), while the non-synesthetic stimulus was a symbol created from the trait features of the respective alphanumeric synesthetic stimulus (see Fig. 3). The number “5” was chosen because it evokes a vivid synesthetic sensation of colour in all participants. For synesthete participant #10, the synesthetic stimuli were replaced by a letter “d”, as this participant experienced no synesthesia with digits. The size of the stimuli was 1° × 0.6°. Stimuli were presented on a black square. The suppressor stimulus (1° × 1°) was a visual noise composed of random grains, ranging from black to white. These stimuli were presented side by side at a distance of 3° from the central fixation point of the screen (see Fig. 4).

Stimuli were generated and controlled with Psykinematix sofware, version 1.5 (KyberVision, Sendai, Japan). They were presented dichoptically using 3D glasses (head-mounted virtual-reality display model Z800 3D-Visor; eMagin Corp, Bellevue, WA) driven by a MAC G4 Desktop with an NVIDIA graphics card (GeForce 9400M, Santa Clara, CA). The resolution of each monocular, organic light emitting diode (OLED) screen was 800 by 600 pixels. In each OLED, the refresh rate was 60 Hz and the visual field was 32° by 23°. The size of a pixel subtended an angle of 144 arc/s (0.04°).

Synesthetic and non-synesthetic stimuli, that is, the alphanumeric and abstract stimuli, respectively, were displayed in two experimental conditions. In the achromatic condition, stimuli were presented in grey scale (RGB values = 160). In the chromatic condition, stimuli were displayed with the colour that corresponded to the personal synesthetic perception of each synesthete. The RGB values of the images were then adjusted in order to reach physical equiluminance with the achromatic stimuli (~ 49 cd/m^2^). All stimuli in all conditions were presented on a black background (0.01 cd/m^2^). The stimuli covered approximately 70% of the surface of the background frame. The contrast level was 50%. Participants in the control group were tested with the same stimuli as their corresponding synesthete participant.

Four stimulus sets were generated according to the condition (achromatic and chromatic) and stimulus type (synesthetic and non-synesthetic): alphanumeric achromatic stimulus, achromatic abstract stimulus, alphanumeric chromatic stimulus, and abstract chromatic stimulus. Stimuli were displayed randomly in 3 blocks, each comprising 28 flash suppression trials. In the flash suppression trials, the presentation of the stimuli was dichoptic (see Fig. 4). A pair of suppressor stimuli (visual noise patches) was abruptly presented to the previously-unstimulated eye 3 to 4 s after the stimulus onset (time 0). As a result, the initial pair of target stimuli disappeared from awareness. Thus, the presentation of the visual noise was perceived by participants as a “flash,” which masked the test stimulus, even though it was physically present on the screen.

**Figure 5:**
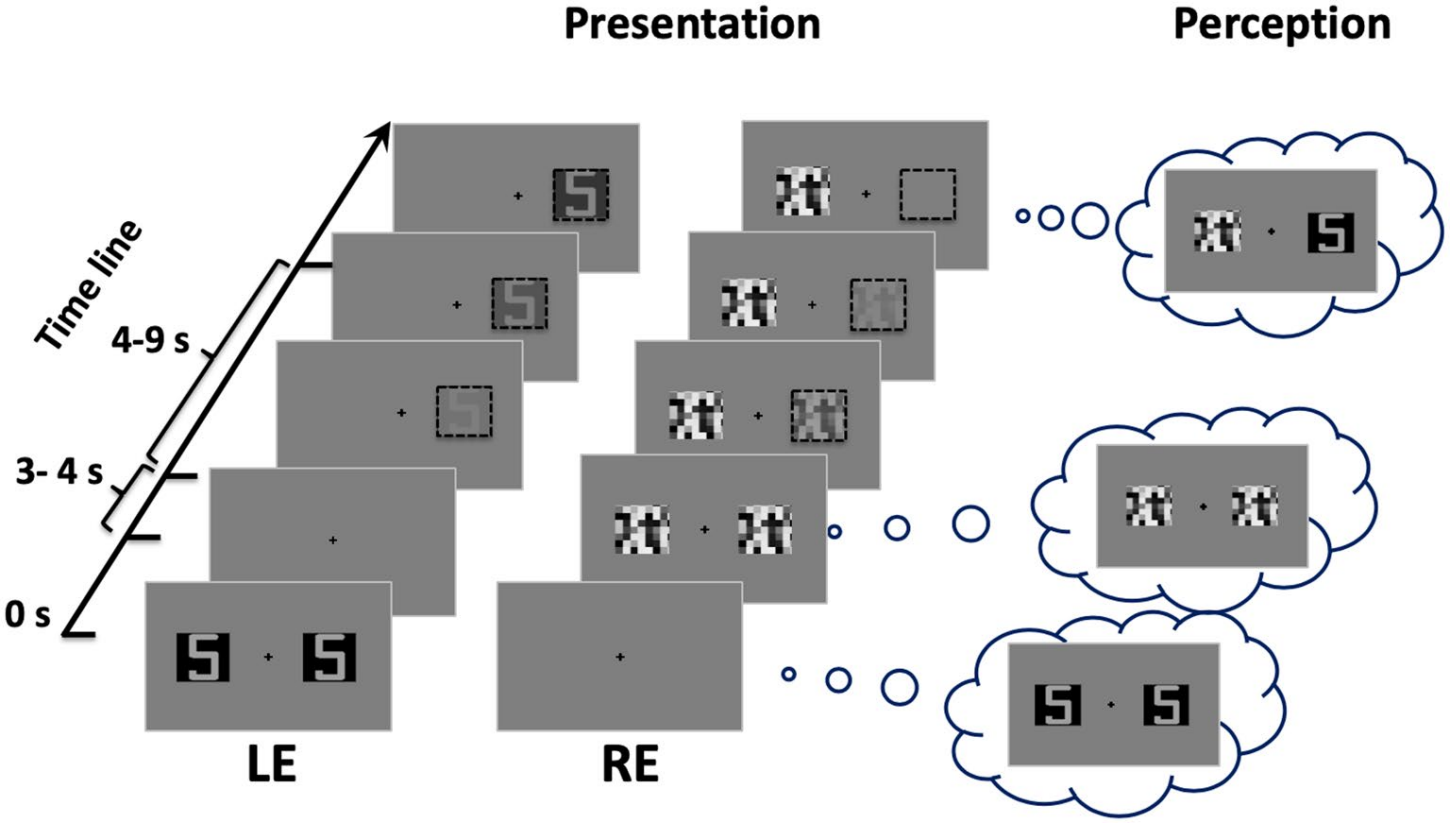
An example of a non-flash-suppression trial. The stimulus presentation was controlled (left side) to mimic the subjective perception (right side) so that one of the two images initially presented reappeared after being suppressed by the sudden presentation of a flash. Dashed-line squares (on the left) illustrate the fade-in/fade-out interplay of the images that were used to mimic the breaking of the flash suppression. LE: left eye; RE: right eye.

In addition to the flash suppression trials, “non-flash-suppression” trials (12 trials per block) were embedded in the task to mimic a stimulus presentation similar to the experimental trials. In the non-flash-suppression trials (see Fig. 5), stimuli were presented in such a way that flash suppression did not occur; the initial stimuli displayed to one eye disappeared after 3–4 s. At the same time the two noise patches were presented to the other eye. After a while, the stimulus target reappeared slowly in one eye (fade-in, from 0 to 50% contrast) while the corresponding noise patch in the other eye disappeared slowly (fade-out, from 50 to 0%). The flash suppression (Fig. 4) and the non-flash-suppression trials (Fig. 5) were randomly presented in each testing block. In addition, stimulus presentation was counterbalanced between the left and the right eyes.

### Procedure

Participants were comfortably seated in a dimly lit room. They were instructed to adjust the alignment of the 3D glasses by moving the lenses sideways and to adjust their proximity. Eight practice trials were performed. The participants were instructed to maintain their gaze on the central fixation dot while the images were presented.

The task method consisted of a spatial two-alternative forced choice. The participants were instructed to press the left or the right arrow key when one stimulus re-appeared in its entirety, either on the left or the right side of the screen. The time required for a participant to report the reappearance of the hidden stimulus was calculated as the duration of suppression in the flash suppression trials. For the non-flash-suppression trials, the time required to detect the fake suppressed stimulus was measured. Both types of trials (flash suppression and non-flash-suppression) ended when participants responded, or after 15 s. Each trial was launched by pressing the “enter” key. Participants were allowed to take breaks between blocks if they desired.

### Data analyses

Intra- and inter-group differences were assessed using analyses of variance (ANOVA) with the condition (achromatic and chromatic), the stimulus (alphanumeric and abstract stimuli), and the group (synesthetes and controls) as the main factors. Separate ANOVA were conducted for the flash suppression and the non-flash-suppression trials. Data were also analyzed using mixed effects modeling to treat subjects as a random factor, while the condition, stimulus and group were fixed. For the flash suppression trials, the duration of suppression was calculated by subtracting the onset of the suppressor stimulus presentation (noisy patches) from the participants’ reaction time. A lower value meant a faster time for the target to reach visual awareness. For the non-flash-suppression trials, the time required to detect the fake suppressed stimulus was calculated from the fade-in onset of the stimulus target to the reaction time of the participant. A lower value meant that the stimulus was rapidly detected. In all analyses, the *p* values were set to be significant at an α level of < 0.05. Bonferroni corrections were applied to detect the significance in post-hoc pairwise comparisons. ANOVA and mixed effects modeling were analyzed using IBM SPSS Statistics (version 24.0). Additional Bayesian statistical analyses were conducted with JASP package (version 0.14.1)^25^ to weigh evidence for null versus alternative hypotheses. Bayesian ANOVAs were conducted using JASP default priors, and effects are reported as the Bayes factor for the inclusion of a particular effect, calculated as the ratio between the likelihood of the data given the model with vs the next simpler model without that effect.

## Supplementary Information

Supplementary Information.
