# Supplementary material for: Synesthesia does not help to recover perceptual dominance following flash suppression
Source: Sci Rep. 2021 Apr 7;11:7566. doi: 10.1038/s41598-021-87223-w (PMC8027846; doi:10.1038/s41598-021-87223-w)
Supplement: Supplementary file 1 — Supplementary Information. [file 41598_2021_87223_MOESM1_ESM.docx]

**Synesthesia does not help to recover perceptual dominance following flash suppression**

Diana Jimena Arias ^1,2^ and Dave Saint-Amour **^1,2,3,*^**

^1^Department of Psychology, Université du Québec à Montréal, Montréal, H2X 3P2, Canada

^2^Cognitive Neurosciences Research Center, Université du Québec à Montréal, Montréal, H2X 3P2, Canada

^3^Research Center of the Sainte-Justine University Hospital, Montréal, H3T 1C5, Canada

* Corresponding author**:**
E-mail: saint-amour.dave@uqam.ca

**Supplementary Table S1.** Results for flash suppression trials obtained from mixed effects modeling. Duration of suppression was the dependent variable; Cond (Cond), Stim (Stim) and Group (Gr) were the independent variables; Subject was the random effect.

| **Fixed effects** | **nDf** | **dDf** | ***F* value** | ***p* value** |
| --- | --- | --- | --- | --- |
| *Main effects* |  |  |  |  |
| Gr | 1 | 19 | .52 | .478 |
| Cond | 1 | 57 | 75.06 | < .0001 |
| Stim | 1 | 57 | 33.25 | < .0001 |
|  |  |  |  |  |
| *Interactions* |  |  |  |  |
| Gr*Cond | 1 | 57 | .32 | .569 |
| Gr*Stim | 1 | 57 | 1.10 | .298 |
| Cond*Stim | 1 | 57 | 10.49 | .002 |
| Gr*Cond* Stim | 1 | 57 | .00 | .987 |

**Supplementary Table S2.** Results for non-flash suppression trials obtained from mixed effects modeling. Duration of suppression was the dependent variable; Cond (Cond), Stim (Stim) and Group (Gr) were the independent variables; Subject was the random effect.

| **Fixed effects** | **nDf** | **dDf** | ***F* value** | ***p* value** |
| --- | --- | --- | --- | --- |
| *Main effects* |  |  |  |  |
| Gr | 1 | 19 | 5.651 | .028 |
| Cond | 1 | 57 | 29.522 | < .0001 |
| Stim | 1 | 57 | 26.269 | < .0001 |
|  |  |  |  |  |
| *Interactions* |  |  |  |  |
| Gr*Cond | 1 | 57 | 3.698 | .059 |
| Gr*Stim | 1 | 57 | 3.153 | .081 |
| Cond*Stim | 1 | 57 | .296 | .588 |
| Gr*Cond* Stim | 1 | 57 | 1.147 | .289 |

**Supplementary Table S3.** Hierarchical model comparison obtained from the Bayesian repeated measures ANOVA for flash suppression trials. Results were computed using JASP program.

| **Model Comparison** | | | | | |
| --- | --- | --- | --- | --- | --- |
| *Models* | *P(M)* | *P(M\|data)* | *BF _M_* | *BF_10_* | *error %* |
| Null model (incl. subject) | .053 | 1.950e -12 | 3.510e-11 | 1 |  |
| Cond+ Stim+ Cond ✻ Stim | .053 | .402 | 12.089 | 2.061e+11 | 10.184 |
| Cond + Stim + Gr+ Cond ✻ Stim | .053 | .27 | 6.646 | 1.383e+11 | 2.629 |
| Cond + Stim + Gr + Cond ✻ Stim + Stim ✻ Gr | .053 | .122 | 2.508 | 6.272e+10 | 2.264 |
| Cond + Stim + Gr + Cond ✻ Stim + Cond   ✻ Gr | .053 | .092 | 1.816 | 4.699e+10 | 4.315 |
| Cond + Stim + Gr + Cond ✻ Stim + Cond ✻ Gr + Stim ✻ Gr | .053 | .046 | .873 | 2.373e+10 | 6.47 |
| Cond + Stim | .053 | .023 | .416 | 1.159e+10 | 2.122 |
| Cond + Stim + Gr | .053 | .016 | .29 | 8.120e+9 | 1.736 |
| Cond + Stim + Gr + Cond ✻ Stim + Cond ✻ Gr + Stim ✻ Gr + Cond ✻ Stim ✻ Gr | .053 | .015 | .278 | 7.801e+9 | 3.76 |
| Cond + Stim + Gr + Stim ✻ Gr | .053 | .007 | .127 | 3.580e+9 | 3.075 |
| Cond + Stim + Gr + Cond ✻ Gr | .053 | .006 | .1 | 2.837e+9 | 2.942 |
| Cond + Stim + Gr + Cond ✻ Gr + Stim ✻ Gr | .053 | .002 | .04 | 1.140e+9 | 2.53 |
| Cond | .053 | 2.592e-6 | 4.665e -5 | 1.329e +6 | 3.176 |
| Cond + Gr | .053 | 1.807e-6 | 3.253e -5 | 926871.016 | 2.899 |
| Cond + Gr + Cond ✻ Gr | .053 | 5.448e-7 | 9.807e-6 | 279434.816 | 1.81 |
| Stim | .053 | 1.278e -10 | 2.301e-9 | 65.563 | 1.658 |
| Stim + Gr | .053 | 8.012e-11 | 1.442e-9 | 41.093 | 1.304 |
| Stim + Gr + Stim ✻ Gr | .053 | 2.954e -11 | 5.317e -10 | 15.15 | 3.254 |
| Gr | .053 | 1.209e -12 | 2.177e -11 | .62 | .599 |

**Supplementary Table S4.** Effects obtained from the Bayesian repeated measures ANOVA for flash suppression trials. Evidence (BF_incl_) was found in favor of an effect of Condition (Cond), Stimulus (Stim) and their interaction (Cond*Stim). There was no conclusive evidence for or against an main effect or interaction of Group. Results were computed using JASP program.

| **Analysis of Effects** | | | |
| --- | --- | --- | --- |
| *Effects* | *P(incl)* | *P(incl\|data)* | *BF_incl_* |
| Cond | .263 | .045 | 1.887e+8 |
| Stim | .263 | .044 | 8892.142 |
| Gr | .263 | .285 | .673 |
| Cond ✻ Stim | .263 | .932 | 17.523 |
| Cond ✻ Gr | .263 | .146 | .351 |
| Stim ✻ Gr | .263 | .178 | .465 |
| Cond ✻ Stim ✻ Gr | .053 | .015 | .329 |

**Supplementary Table S5.** Model comparison obtained from the Bayesian repeated measures ANOVA for non-flash suppression trials. Results were computed using JASP program.

| **Model Comparison** | | | | | |  |
| --- | --- | --- | --- | --- | --- | --- |
| *Models* | *P(M)* | *P(M\|data)* | *BF_M_* | *BF_10_* | *Error %* |  |
| Null model (incl. subject) | .053 | 3.302e-8 | 5.944e-7 | 1 |  | |
| Cond + Stim + Gr + Cond ✻ Gr + Stim ✻ Gr | .053 | .202 | 4.545 | 6.105e+6 | 3.224 |  |
| Cond + Stim + Gr + Cond ✻ Gr | .053 | .185 | 4.082 | 5.598e+6 | 5.999 |  |
| Cond + Stim + Gr + Stim ✻ Gr | .053 | .139 | 2.914 | 4.220e+6 | 7.646 |  |
| Cond + Stim + Gr | .053 | .138 | 2.88 | 4.177e+6 | 3.717 |  |
| Cond + Stim + Gr + Cond ✻ Stim + Cond ✻ Gr + Stim ✻ Gr | .053 | .064 | 1.24 | 1.952e+6 | 7.106 |  |
| Cond + Stim | .053 | .061 | 1.164 | 1.839e+6 | 1.483 |  |
| Cond + Stim + Gr + Cond ✻ Stim + Cond ✻ Gr | .053 | .058 | 1.104 | 1.750e+6 | 4.965 |  |
| Cond + Stim + Gr + Cond ✻ Stim + Stim ✻ Gr | .053 | .053 | 1.003 | 1.598e+6 | 3.385 |  |
| Cond + Stim + Gr + Cond ✻ Stim | .053 | .046 | .869 | 1.394e+6 | 3.496 |  |
| Cond + Stim + Gr + Cond ✻ Stim + Cond ✻ Gr + Stim ✻ Gr + Cond ✻ Stim ✻ Gr | .053 | .032 | .594 | 967399.73 | 4.124 |  |
| Cond + Stim + Cond ✻ Stim | .053 | .022 | .412 | 677007.5 | 8.814 |  |
| Cond + Gr | .053 | 5.192e-5 | 9.346e-4 | 1572.302 | 3.715 |  |
| Cond + Gr + Cond ✻ Gr | .053 | 4.586e-5 | 8.256e-4 | 1388.945 | 2.159 |  |
| Cond | .053 | 2.097e-5 | 3.775e-4 | 635.137 | 1.198 |  |
| Stim + Gr | .053 | 1.967e-5 | 3.540e-4 | 595.62 | 4.056 |  |
| Stim + Gr + Stim ✻ Gr | .053 | 1.260e-5 | 2.269e-4 | 381.665 | 2.307 |  |
| Stim | .053 | 7.542e-6 | 1.357e-4 | 228.386 | 1.184 |  |
| Gr | .053 | 9.579e-8 | 1.724e-6 | 2.901 | 12.713 |  |

**Supplementary Table S6.** Effects obtained from the Bayesian repeated measures ANOVA for non-flash suppression trials. Evidence (BF_incl_) was found in favor of an effect of Condition (Cond), Stimulus (Stim) and their interaction (Cond*Stim). There was no conclusive evidence for or against an main effect or interaction of Group. Results were computed using JASP program.

| **Analysis of Effects** | | | | |
| --- | --- | --- | --- | --- |
| *Effects* | *P(incl)* | *P(incl\|data)* | *BF_incl_* |  |
| Cond | .263 | .338 | 8464.645 |  |
| Stim | .263 | .384 | 3226.375 |  |
| Gr | .263 | .184 | 2.214 |  |
| Cond ✻ Stim | .263 | .243 | .336 |  |
| Cond ✻ Gr | .263 | .509 | 1.353 |  |
| Stim ✻ Gr | .263 | .458 | 1.074 |  |
| Cond ✻ Stim ✻ Gr | .053 | .032 | .496 |  |
